# A Rare Head and Neck Tumor: Making Simple Things Complicated

**DOI:** 10.7759/cureus.76576

**Published:** 2024-12-29

**Authors:** Chintamani Chintamani, Shagun Agarwal, Sakura Shrestha, Shashi Dhawan

**Affiliations:** 1 Surgical Oncology, Sir Ganga Ram Hospital, New Delhi, IND; 2 Pathology, Sir Ganga Ram Hospital, New Delhi, IND

**Keywords:** cutaneous t cell lymphoma, definitive treatment, onco dermatology, r-chop therapy, surgical dermatology

## Abstract

Primary cutaneous anaplastic large cell lymphoma (ALCL) is a very uncommon type of CD30-positive T-cell lymphoma, and it very rarely affects the forehead. We report the case of a 68-year-old male presenting with an ulcerative lesion on the right forehead, initially suspected as a benign condition. Fine needle aspiration suggested a lymphoproliferative disorder, with biopsy and immunohistochemistry confirming primary cutaneous ALCL (CD30-positive, anaplastic lymphoma kinase [ALK]-negative). The patient was treated with a standardized chemotherapy regimen and achieved a complete response. This case underscores the importance of considering primary cutaneous ALCL in the differential diagnosis of persistent ulcerative lesions in anatomically sensitive areas. Early diagnosis, multidisciplinary management, and advanced therapeutic strategies such as Brentuximab Vedotin + Cyclophosphamide, Hydroxyrubicin, and Prednisone (BV + CHP) are critical to optimizing outcomes in this rare presentation.

## Introduction

Anaplastic large cell lymphomas (ALCL) represent a distinct subtype of CD30-positive non-Hodgkin T-cell lymphomas, accounting for approximately 3% of all non-Hodgkin lymphomas [1]. Within this group, primary cutaneous ALCL is a rare variant that originates in the skin without evidence of systemic involvement at diagnosis. It belongs to the spectrum of CD30-positive lymphoproliferative disorders, which also includes lymphomatoid papulosis [2]. Together, these disorders represent the second most common group of cutaneous T-cell lymphomas, accounting for 25% of cases [3].

Cutaneous ALCL typically presents as solitary or localized nodules that may ulcerate and mimic benign dermatological conditions such as pyogenic granuloma, infectious processes, or other skin malignancies [4,5]. Its clinical presentation can lead to diagnostic delays, which may negatively affect outcomes in rare cases of systemic dissemination. Advanced-stage disease, although uncommon, carries a poor prognosis [6].

Histologically, cutaneous ALCL is characterized by the presence of large, atypical cells expressing CD30, a transmembrane receptor that is a critical diagnostic and therapeutic target [7]. Despite its indolent course, approximately 10-20% of cases can progress to systemic disease, necessitating comprehensive staging at diagnosis [8]. PET-CT plays a pivotal role in identifying occult systemic involvement and guiding therapeutic decisions.

Therapeutic strategies for primary cutaneous ALCL have evolved significantly in recent years. The advent of antibody-drug conjugates such as Brentuximab Vedotin (BV) has improved clinical outcomes. The BV + CHP (Cyclophosphamide, Hydroxyrubicin, Prednisone) regimen has demonstrated superior progression-free survival and response rates compared to conventional CHOP (Cyclophosphamide, Hydroxyrubicin, Oncovin, Prednisone) chemotherapy, making it the preferred regimen for CD30-positive ALCL [9,10]. This case highlights the importance of considering primary cutaneous ALCL in the differential diagnosis of persistent nodular and ulcerative lesions, especially in anatomically sensitive locations such as the forehead.

## Case presentation

**Figure 1:**
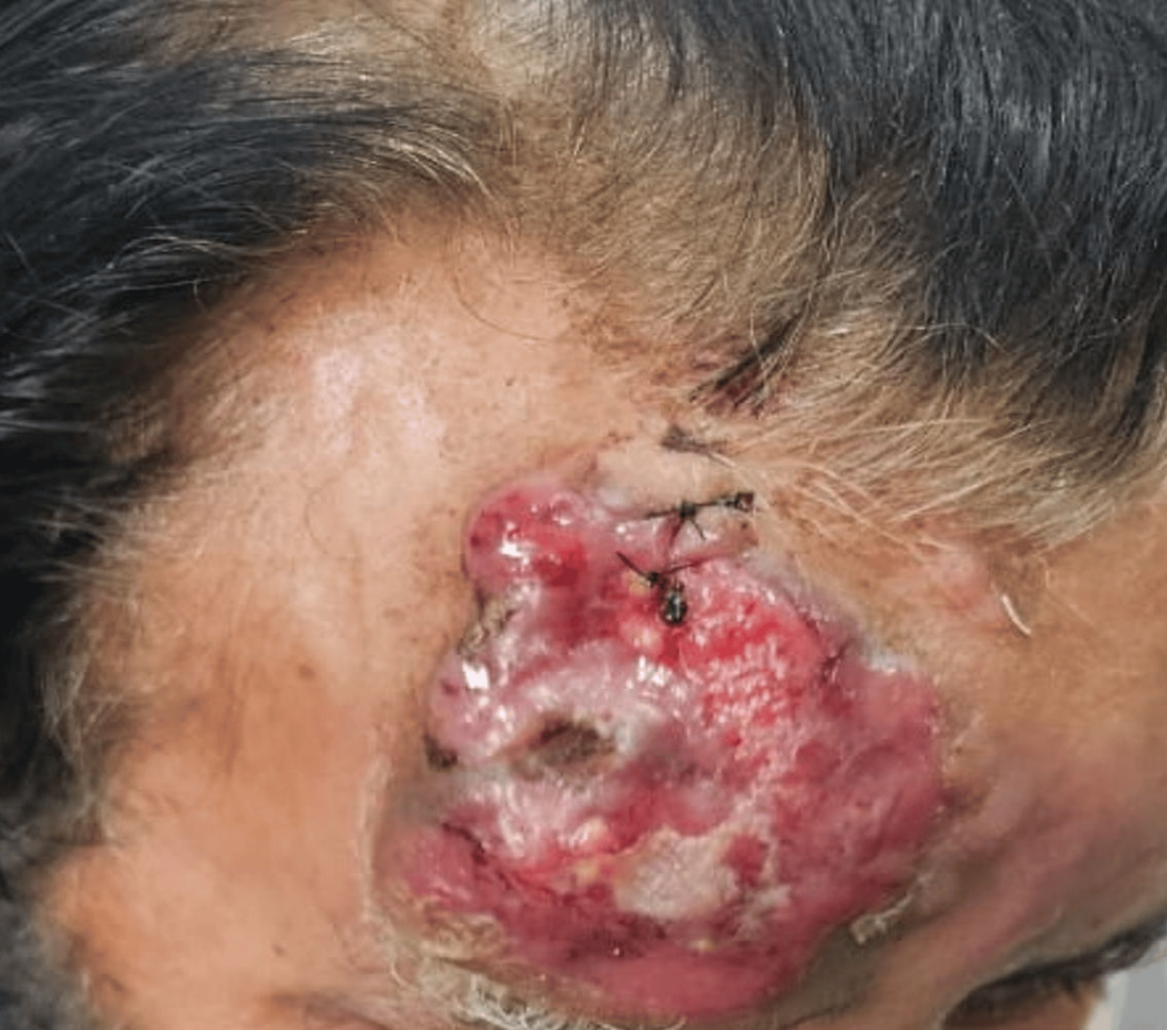
At the time of presentation

**Figure 2:**
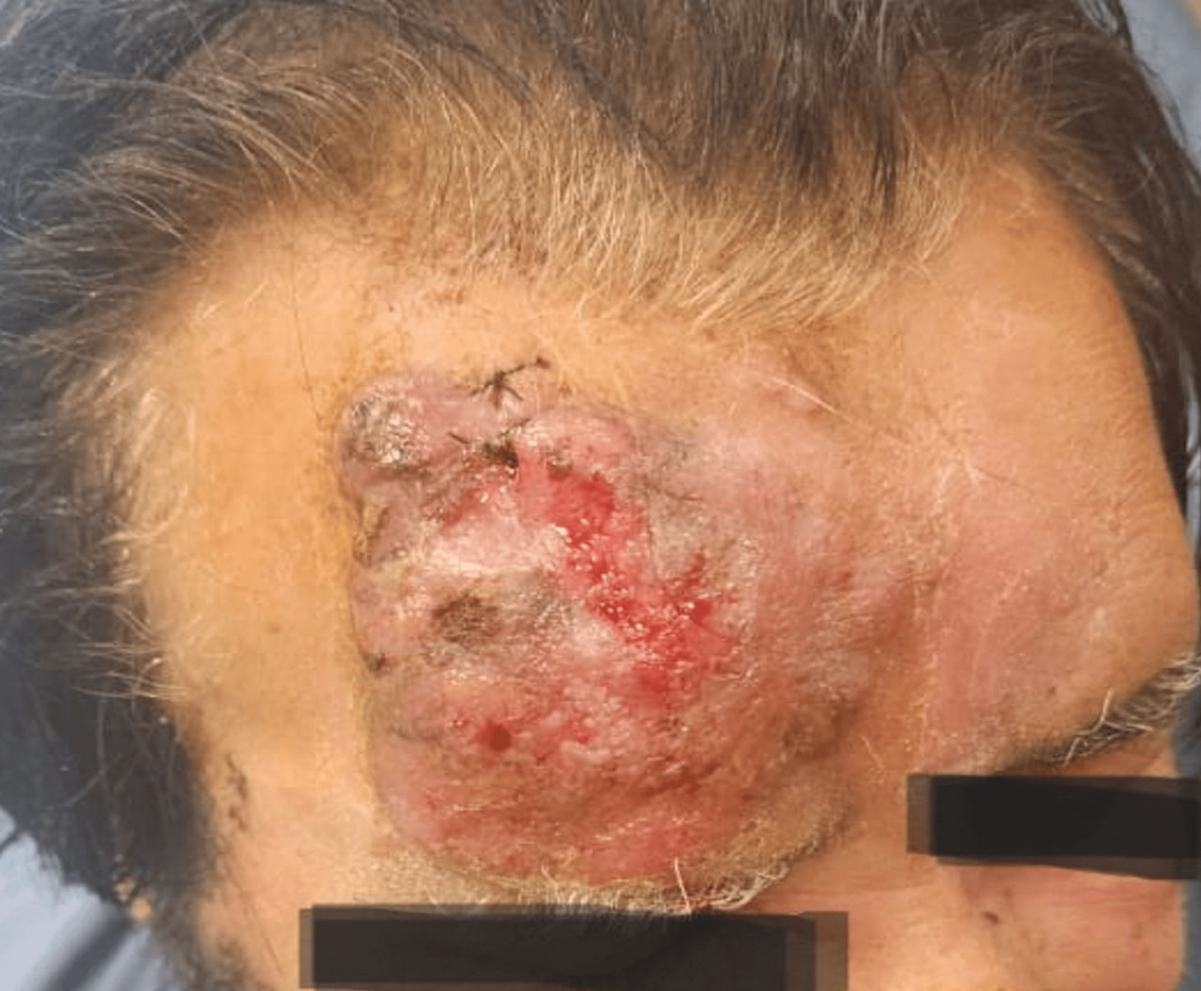
After second cycle of chemotherapy

A 68-year-old gentleman presented to us with swelling over his forehead above his right eyebrow. He was being followed up by a dermatologist and was referred to the Department of Surgery upon recent ulceration of the swelling for wide local excision and reconstruction. The patient searched the internet for a head and neck surgeon as he was convinced that he would have to undergo the procedure. He landed up with us when a detailed history and examination were undertaken. There was no history of any immunosuppressive drug intake or disorder. The swelling was 5x5 cm in size in the right supraorbital region, extending up to the scalp with overlying skin ulceration (Figures 1, 2).

**Figure 3:**
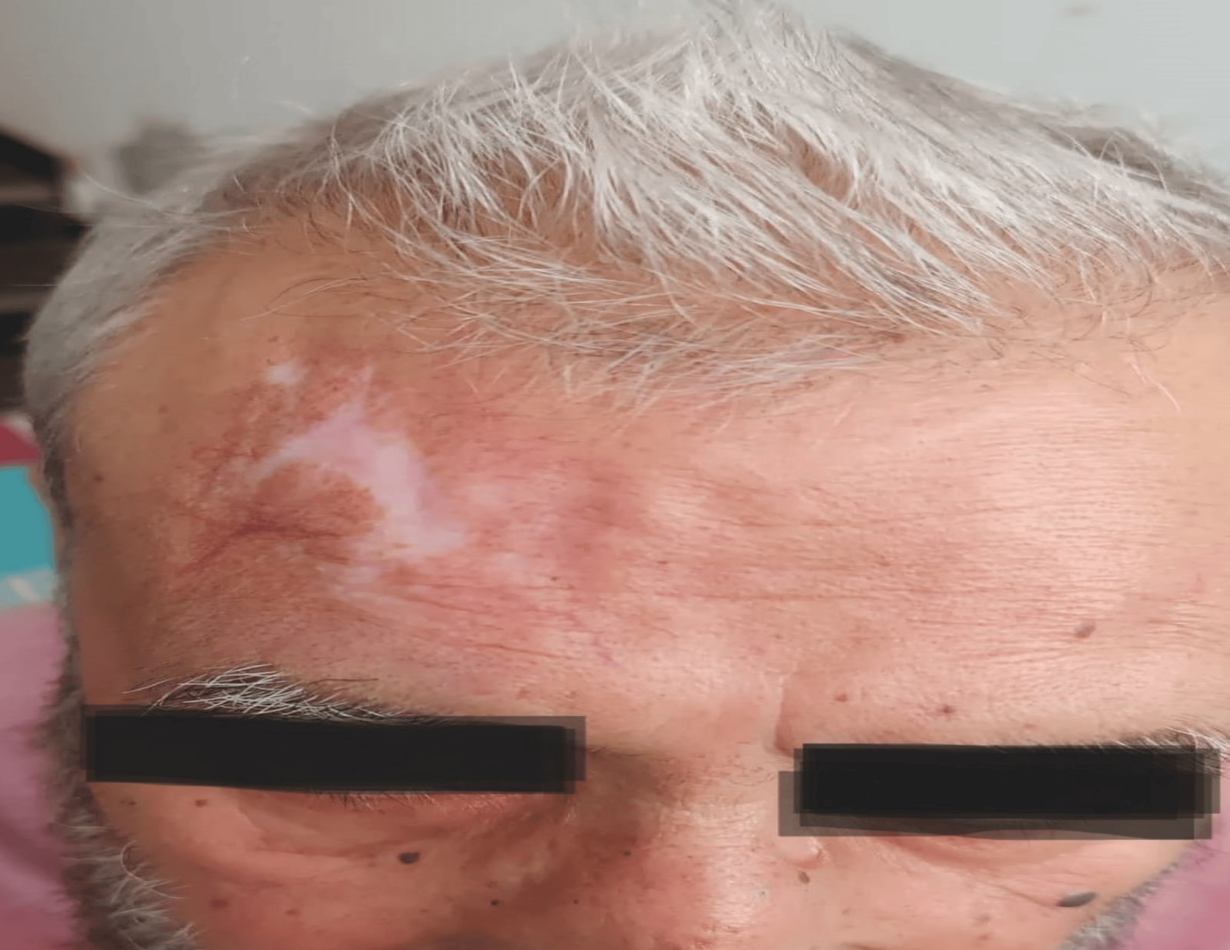
Healed scar after sixth cycle with no residual disease on PET

Fine needle aspiration (FNA) was suggestive of a lymphoproliferative disorder, following which a wedge biopsy was performed from the superior edge of the lesion that revealed “a non-Hodgkin lymphoma, T-phenotype compatible with primary cutaneous anaplastic large cell lymphoma.” On IHC, the cells were diffusely positive for CD3 and CD30 and negative for CD20, ALK1, CD5, GATA3, CD10, BCL6, and CD56. Ki-67 proliferative index was 60-70%. Metastatic workup in the form of PET CT was suggestive of multiple FDG-avid, heterogeneously enhancing, discrete, and coalescent soft tissue nodular mass lesions arising from the right side of the forehead and right preauricular region. The multidisciplinary meeting decided to put the patient on primary chemotherapy, viz. six cycles of the BV + CHP regimen (Brentuximab Vedotin, Cyclophosphamide, Doxorubicin, and Prednisone), which showed a tremendous response with complete resolution of the ulcer and swelling (Figure 3). The patient is currently on regular follow-up and is in remission.

## Discussion

Primary cutaneous ALCL is a rare malignancy, with the head and neck region being the most commonly involved site. However, cases involving the forehead are particularly unusual and sparsely reported [4,5]. This unique anatomical site raises additional challenges due to the proximity of critical structures, including the eyes and central nervous system, increasing the risk of functional complications such as vision impairment.

**Figure 4:**
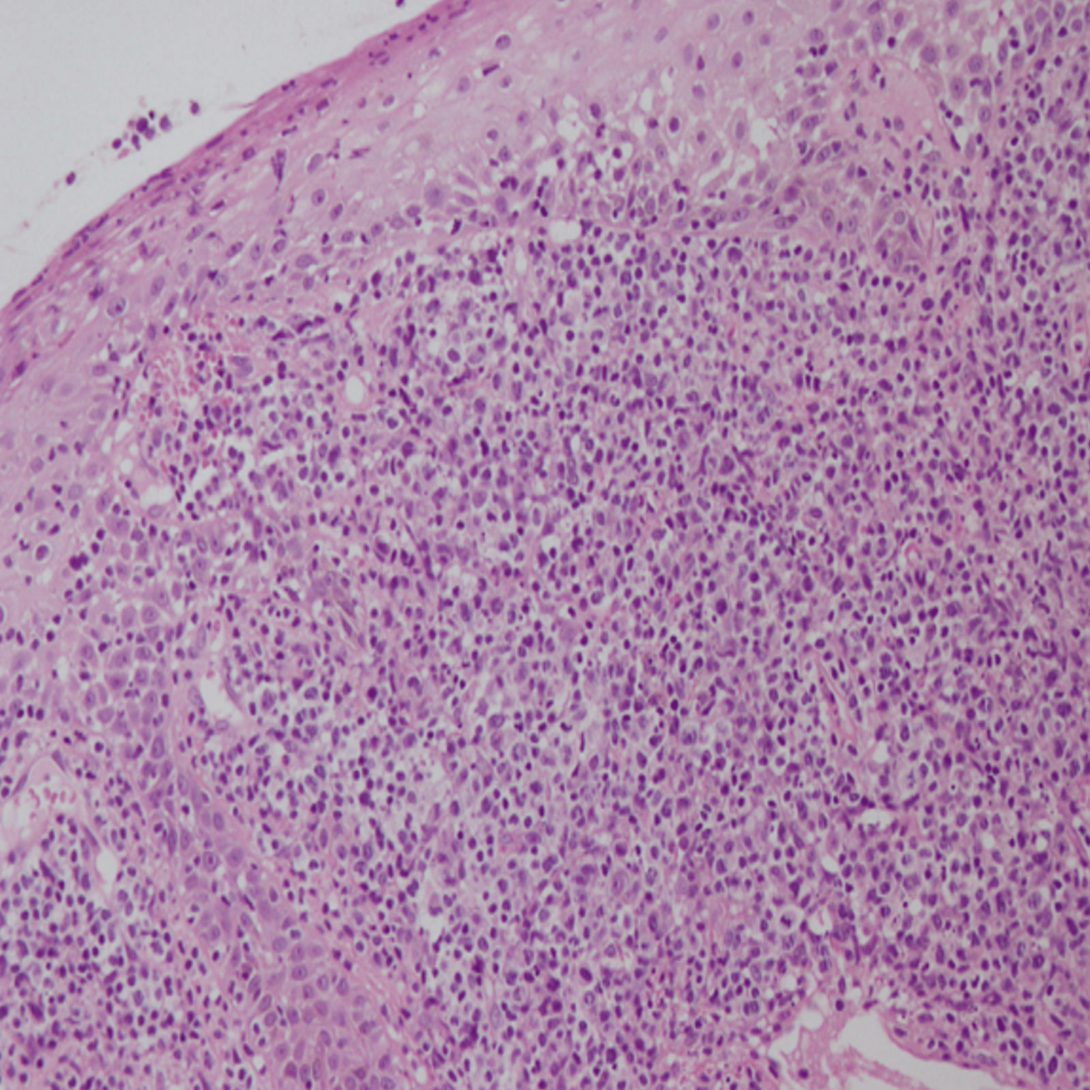
Cohesive sheets of large atypical lymphoid cells diffusely infiltrating into the dermis while focally into the epidermis. These cells have irregularly shaped nuclei and abundant cytoplasm. Mitosis is high. Magnification 200x (H&E)

**Figure 5:**
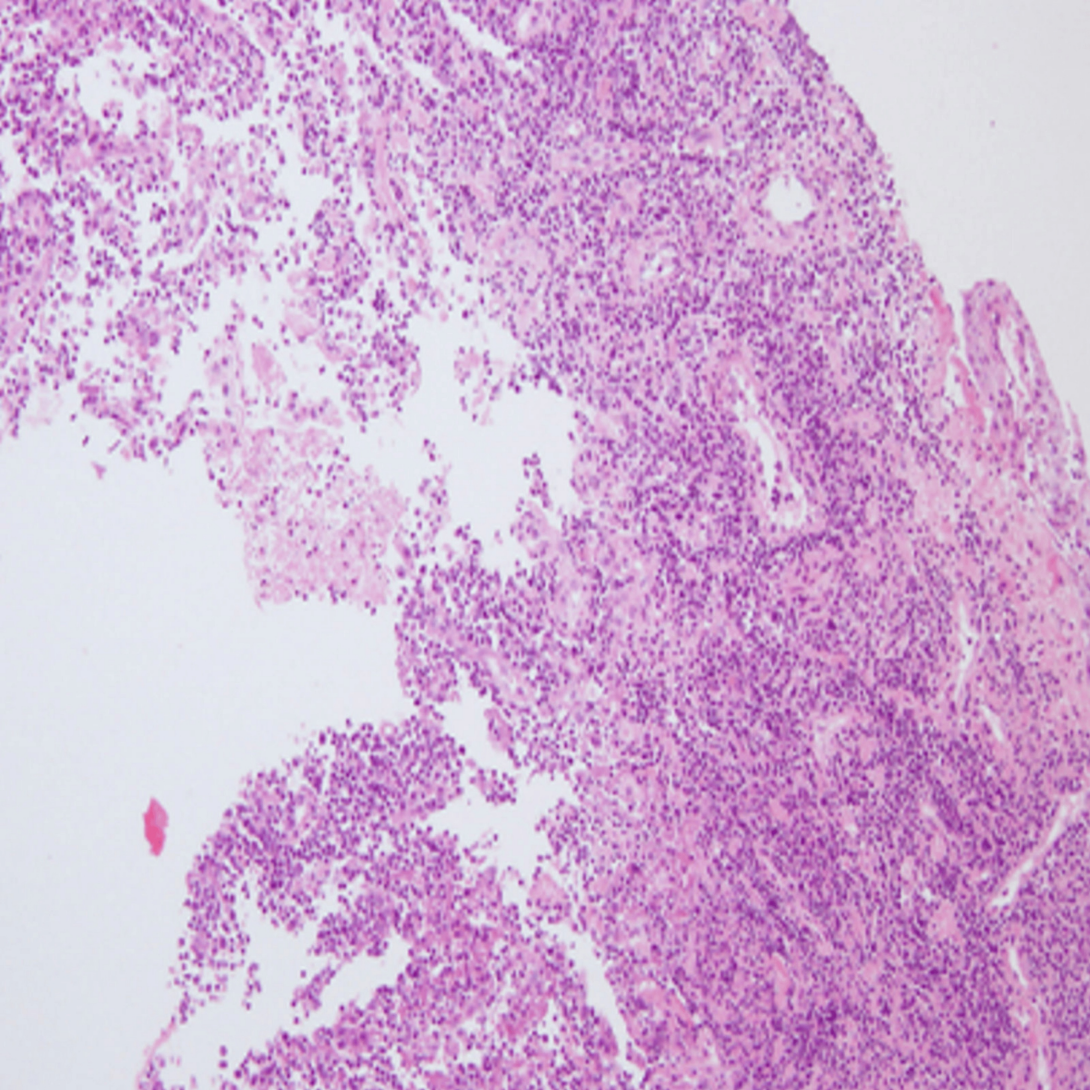
Showing ulceration of skin with focal necrosis. Abundant mixed inflammatory infiltrate of reactive T cells, histiocytes, eosinophils, and neutrophils also noted at ulcerated surface. Prominent epidermotropism is seen in the intact epidermis. Magnification 200x (H&E)

**Figure 6:**
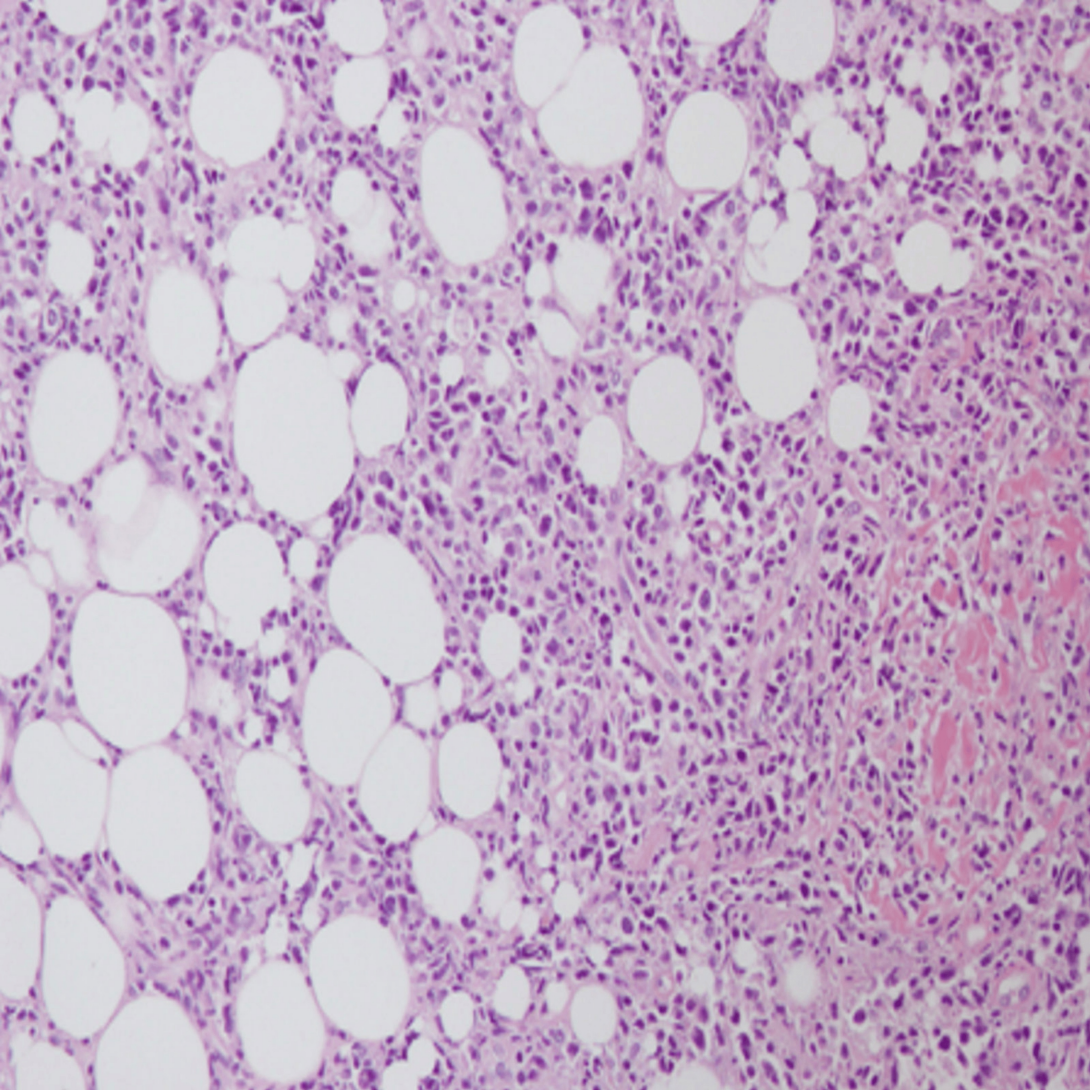
Atypical lymphoid cells infiltrating into the subcutaneous fat. Magnification 200x (H&E)

The differential diagnosis of cutaneous ALCL includes benign conditions such as pyogenic granuloma and infectious or inflammatory processes, as well as malignancies like basal cell carcinoma, squamous cell carcinoma, and metastatic skin tumors [6]. A high index of suspicion is required to ensure timely diagnosis and histopathological confirmation remains the gold standard (Figures 4-6).

The hallmark of primary cutaneous ALCL is the expression of CD30, which is detectable in nearly 100% of cases [7]. Immunohistochemistry further aids in differentiating ALCL from other T-cell lymphomas and cutaneous malignancies.

While primary cutaneous ALCL generally exhibits an indolent course, approximately 10-20% of cases may develop extracutaneous disease [8]. This progression underscores the importance of comprehensive staging with PET-CT, which has high sensitivity for detecting systemic involvement.

The treatment of primary cutaneous ALCL is guided by the extent of the disease. For localized disease, radiotherapy or surgical excision may suffice; however, multifocal or advanced disease requires systemic therapy. The introduction of Brentuximab Vedotin, an anti-CD30 antibody-drug conjugate, has significantly improved outcomes in ALCL [9]. In the ECHELON-2 trial, the BV + CHP regimen demonstrated a 53% improvement in progression-free survival compared to CHOP in CD30-positive peripheral T-cell lymphomas [9]. This case supports these findings, as our patient showed complete remission following six cycles of BV + CHP chemotherapy.

Additionally, CD30 serves as a prognostic biomarker and therapeutic target. Monitoring CD30 levels during follow-up can provide insight into disease status and guide decisions regarding retreatment [10]. Although systemic progression is rare, long-term follow-up remains crucial, particularly in patients with high-risk features such as multifocal disease or ulceration.

Finally, this case underscores the importance of a multidisciplinary approach in managing primary cutaneous ALCL. Coordinated care involving dermatologists, oncologists, and surgical teams ensures optimal outcomes, particularly in rare and anatomically complex presentations.

## Conclusions

Primary cutaneous ALCL is a rare but potentially curable malignancy with favorable long-term outcomes, particularly in the absence of systemic involvement. Early recognition and accurate diagnosis depend on a high index of suspicion and correlation of clinical, histopathological, and imaging findings.

Standardized chemotherapy regimens, such as BV + CHP, form the cornerstone of treatment, while surgery may be necessary for residual lesions. Follow-up protocols focusing on CD30 monitoring are vital for detecting recurrence and guiding further management. This case emphasizes the importance of multidisciplinary care and the need to consider primary cutaneous ALCL in the differential diagnosis of non-healing ulcerative lesions.
